# Supplementary material for: Mapping the evolution of fertility support policies in China: A content and instrumental analysis
Source: PLoS One. 2025 Oct 9;20(10):e0332137. doi: 10.1371/journal.pone.0332137 (PMC12510515; doi:10.1371/journal.pone.0332137)
Supplement: S1 Appendix — (ZIP) [file pone.0332137.s001.zip › S1 Appendix. 226 original policy documents/97-女职工保健工作规定(FBM-CLI-4-9390).doc]

女职工保健工作规定

**发布部门：** [卫生部(已撤销)](https://www.pkulaw.com/law/chl?Aggs.IssueDepartment=60222) [劳动和社会保障部(含劳动部)(已撤销)](https://www.pkulaw.com/law/chl?Aggs.IssueDepartment=60212) [人事部(已撤销)](https://www.pkulaw.com/law/chl?Aggs.IssueDepartment=60211) [中华全国总工会](https://www.pkulaw.com/law/chl?Aggs.IssueDepartment=70401) [全国妇女联合会](https://www.pkulaw.com/law/chl?Aggs.IssueDepartment=70403) [机构沿革](javascript:void(0))

**发文字号：**卫妇发[1993]第11号

**发布日期：**1993.11.26

**实施日期：**1993.11.26

**时效性：** [现行有效](https://www.pkulaw.com/law/chl?Aggs.TimelinessDic=01)

**效力级别：** [部门规章](https://www.pkulaw.com/law/chl?Aggs.EffectivenessDic=XE0301)

**法规类别：** [劳动安全与劳动保护](https://www.pkulaw.com/law/chl?Aggs.Category=08706)

卫生部　劳动部　人事部　全国总工会　全国妇联
关于颁发《女职工保健工作规定》的通知
（卫妇发（1993）第11号）

　　现将根据卫生部、劳动部、人事部、全国总工会、全国妇联1986年联合发布的《女职工保健工作暂行规定（试行草案）》＜86＞卫妇字第7号文修改而成的《女职工保健工作规定》发给你们，望认真贯彻执行，《女职工保健工作暂行规定（试行草案）》同时废止。
　　　　　　　　　　　　　　　　　　　　　1993年11月26日
　　　　　　　　　　　　　 女职工保健工作规定

第一章　总则

　　**第一条**　为保护女职工的身心健康及其子女的健康发育和成长，提高民族素质，根据《[中华人民共和国妇女权益保障法](https://www.pkulaw.com/chl/dff2eefbddf155b3bdfb.html)》和《[女职工劳动保护规定](https://www.pkulaw.com/chl/133408887a349cdabdfb.html)》，特制定本规定。

　　**第二条**　女职工保健工作必须贯彻预防为主的方针，注意女性生理和职业特点，认真执行国家有关保护女职工的各项政策和法规。

　　**第三条**　本规定适用于中华人民共和国境内的一切党政机关、人民团体和企业、事业单位。

第二章　组织措施

　　**第四条**　本规定由各单位分管女职工保健工作的行政领导负责组织本单位医疗卫生、劳动、人事部门和工会、妇联组织及有关人员共同实施。

　　**第五条**　县（含城市区）以上的各级妇幼保健机构，负责对管辖范围内的各单位实施本规定进行业务指导。

　　**第六条**　各单位的医疗卫生部门应负责本单位女职工保健工作。女职工人数在1000人以下的厂矿应设兼职妇女保健人员；女职工人数在1000人以上的厂矿，在职工医院的妇产科或妇幼保健站中应有专人负责女职工保健工作。

第三章　保健措施

　　**第七条**　月经期保健

　　1．宣传普及月经期卫生知识。

　　2．女职工在100人以上的单位，应逐步建立女职工卫生室，健全相应的制度并设专人管理，对卫生室管理人员应进行专业培训。女职工每班在100人以下的单位，应设置简易的温水箱及冲洗器。对流动、分散工作单位的女职工应发放单人自用冲洗器。

　　3．女职工在月经期间不得从事《女职工禁忌劳动范围的规定》中第四条所规定的作业。

　　4．患有重度痛经及月经过多的女职工，经医疗或妇幼保健机构确诊后，月经期间可适当给予1至2天的休假。

　　**第八条**　婚前保健

　　对欲婚女职工必须进行婚前卫生知识的宣传教育及咨询，并进行婚前健康检查及指导。

　　**第九条**　孕前保健

　　1．已婚待孕女职工禁忌从事铅、汞、苯、镉等作业场所属于《有毒作业分级》标准中第Ⅲ－Ⅳ级的作业。

　　2．积极开展优生宣传和咨询。

　　3．对女职工应进行妊娠知识的健康教育，使她们在月经超期时主动接受检查。

　　4．患有射线病、慢性职业中毒、近期内有过急性中毒史及其它有碍于母体和胎儿健康疾病者，暂时不宜妊娠。

　　5．对有过两次以上自然流产史，现又无子女的女职工，应暂时调离有可能直接或间接导致流产的作业岗位。

　　**第十条**　孕期保健

　　1．自确立妊娠之日起，应建立孕产妇保健卡（册），进行血压、体重、血、尿常规等基础检查。对接触铅、汞的孕妇，应进行尿中铅、汞含量的测定。

　　2．定期进行产前检查、孕期保健和营养指导。

　　3．推广孕妇家庭自我监护，系统观察胎动、胎心、宫底高度及体重等。

　　4．实行高危孕妇专案管理，无诊疗条件的单位应及时转院就诊，并配合上级医疗和保健机构严密观察和监护。

　　5．女职工较多的单位应建立孕妇休息室。妊娠满7个月应给予工间休息或适当减轻工作。

　　6．妊娠女职工不应加班加点，妊娠7个月以上（含7个月）一般不得上夜班。

　　7．女职工妊娠期间不得从事劳动部颁布的《女职工禁忌劳动范围的规定》第六条所规定的作业。

　　8．从事立位作业的女职工，妊娠满7个月后，其工作场所应设立工间休息座位。

　　9．有关女职工产前、产后、流产的假期及待遇按1988年国务院颁发的《[女职工劳动保护规定](https://www.pkulaw.com/chl/133408887a349cdabdfb.html)》（国务院令第9号）和1988年劳动部《[关于女职工生育待遇若干问题的通知](https://www.pkulaw.com/chl/2bb380c15892a7f3bdfb.html)》（劳险字＜1988＞2号）执行。

　　**第十一条**　产后保健

　　1．进行产后访视及母乳喂养指导。

　　2．产后42天对母子进行健康检查。

　　3．产假期满恢复工作时，应允许有1至2周时间逐渐恢复原工作量。

　　**第十二条**　哺乳期保健

　　1．宣传科学育儿知识，提倡4个月内纯母乳喂养。

　　2．对有未满1周岁婴儿的女工，应保证其授乳时间。

　　3．婴儿满周岁时，经县（区）以上（含县、区）医疗或保健机构确诊为体弱儿，可适当延长授乳时间，但不得超过6个月。

　　4．有未满1周岁婴儿的女职工，一般不得安排上夜班及加班、加点。

　　5．有哺乳婴儿5名以上的单位，应逐步建立哺乳室。

　　6．不得安排哺乳女职工从事《[女职工劳动保护规定](https://www.pkulaw.com/chl/133408887a349cdabdfb.html)》和《女职工禁忌劳动范围的规定》所指出的作业。

　　**第十三条**　更年期保健

　　1．宣传更年期生理卫生知识，使进入更年期的女职工得到社会广泛的关怀。

　　2．经县（区）以上（含县、区）的医疗或妇幼保健机构诊断为更年期综合症者，经治疗效果仍不显著，且不适应原工作的，应暂时安排适宜的工作。

　　3．进入更年期的女职工应每1至2年进行一次妇科疾病的查治。

　　**第十四条**　对女职工定期进行妇科疾病及乳腺疾病的查治。

　　**第十五条**　女职工浴室要淋浴化。厕所要求蹲位。

　　**第十六条**　建立健全女职工保健工作统计制度。

第四章　监督管理

　　**第十七条**　各级卫生行政部门会同同级劳动、人事部门，工会及妇联组织对本规定的实施情况进行监督。

　　**第十八条**　凡违反本规定第七条第3款第（1）、（2）、（3）项、第十条第7、9款、第十二条第2、6款的单位负责人或直接责任者，可依据《[女职工劳动保护规定](https://www.pkulaw.com/chl/133408887a349cdabdfb.html)》第[十三条](https://www.pkulaw.com/chl/133408887a349cdabdfb.html#tiao_13)规定进行处理。

　　**第十九条**　凡违反本规定其它条款的单位或直接责任者，各级卫生行政部门可根据情节给予警告、通报批评、限期改进的处罚。

　　**第二十条**　女职工违反国家有关计划生育规定的，其女职工的保健应当按照国家有关计划生育规定办理。

第五章　附则

　　**第二十一条**　本规定中所称企业，系指全民、城镇集体企业，中外合资、合作、独资企业，乡镇企业，农村联户企业、私人企业等。

　　**第二十二条**　女职工包括单位固定女职工、合同制女职工、临时女职工。

　　**第二十三条**　本规定由中华人民共和国卫生部负责解释。

　　**第二十四条**　本规定由颁发之日起实施。

© 北大法宝：（[www.pkulaw.com](https://www.pkulaw.com/)) 专业提供法律信息、法学知识和法律软件领域各类解决方案。北大法宝为您提供丰富的参考资料，正式引用法规条文时请与标准文本核对。
欢迎查看所有[产品和服务](https://www.pkulaw.com/helps/2.html)。法宝快讯：[如何快速找到您需要的检索结果？](https://www.pkulaw.com/helps/70.html)[法宝](https://www.pkulaw.com/helps/69.html)V6有何新特色？

扫描二维码阅读原文

**原文链接：**<https://www.pkulaw.com/chl/6bd61140293005a1bdfb.html>
